# Analysis and application of *Bacillus subtilis *sortases to anchor recombinant proteins on the cell wall

**DOI:** 10.1186/2191-0855-1-22

**Published:** 2011-07-21

**Authors:** Hoang Duc Nguyen, Trang Thi Phuong Phan, Wolfgang Schumann

**Affiliations:** 1 Institute of Genetics, University of Bayreuth, D-95445 Bayreuth, Germany; 2 Center for Bioscience and Biotechnology, University of Science, Vietnam National University, 227 Nguyen Van Cu, District 5, Ho Chi Minh City, Vietnam; 3 Laboratory of Molecular Biotechnology, University of Science, Vietnam National University, 227 Nguyen Van Cu, District 5, Ho Chi Minh City, Vietnam

**Keywords:** Sortase, *B. subtilis*, YhcR, YhcS, surface display, microbiorobot

## Abstract

*Bacillus subtilis *codes for two putative sortases, YhcS and YwpE, and two surface proteins, YhcR and YfkN, harboring sorting motifs supposed to be recognized by the putative sortase(s). However, there is no experimental evidence to show a direct link between these sortases and sorting sequences. To study the role of these two putative sortases on displaying YhcR and YfkN on the cell wall, expression of *yhcS *and *ywpE *was analyzed by transcriptional fusions and by Northern blot. It turned out that *yhcS *gene is expressed at a higher level during the late stationary phase from both experiments, while *ywpE *expression is not confirmed in the Northern blot analysis. Next, we constructed *yhcS *and *ywpE *single and double knockout strains and plasmids that express one or both genes to restore the functions of the knockout strains. It could be shown that display of YhcR and YfkN on the surface depended on the presence of YhcS while YwpE seems not to play a major role if any as a sortase. Finally, the putative sorting motif together with a 123-amino-acid spacer derived from YhcR and YfkN designated YhcR123 and YfkN123, respectively, were fused to an α-amylase reporter enzyme. The fusion protein YhcR123-AmyQ could be displayed on the surface at high amounts, while YfkN123-AmyQ could be hardly detected. We conclude that the sortase YhcS can recognize and anchor YhcR on the cell wall. This result further indicates that the YhcR sorting sequence can be used to display recombinant proteins on the surface of *B. subtilis *cells.

## Introduction

Cell surface display of recombinant proteins is usually achieved through a translational fusion of the target protein to one of the naturally occurring surface proteins of the host cell. Display of proteins on the surface of microorganisms, enabled by means of recombinant DNA technology, has become an increasingly used strategy in various applications in microbiology, biotechnology and vaccination (Samuelson et al. 2002; Wernerus and Stahl 2004; Daugherty 2007).

From a practical point of view, Gram-positive bacteria have certain properties that potentially make them more suitable for bacterial surface display applications. First, the surface proteins of Gram-positive bacteria seem to be more permissive for the insertion of extended sequences of foreign proteins that have several hundreds of amino acids, as compared with the different Gram-negative surface proteins (Samuelson et al. 2002). Second, a more obvious advantage of the Gram-positive system is that translocation through only a single membrane is required to achieve proper surface exposure of the heterologous polypeptide, while in the Gram-negative system both translocation through the cytoplasmic membrane and correct integration into the outer membrane are required for surface display. Finally, considering the practical handling of the bacteria, Gram-positive bacteria have the additional advantage of being more rigid, due to the thicker cell wall (Pagan et al. 1999; Samuelson et al. 2002), which thus allows various laboratory procedures without extensive cell lysis (Desvaux et al. 2006).

In Gram-positive bacteria, a class of surface proteins are covalently anchored on the cell wall by a transpeptidase, which has been called sortase (Srt) (Paterson and Mitchell 2004; Ton-That et al. 2004; Marraffini et al. 2006; Clancy et al. 2010). Sortases are positioned at the cytoplasmic membrane via a membrane anchor located either at the N- or C-terminus, contain the active site, LxTC motif (conserved residues underlined) (Marraffini et al. 2006), of which cystein is essential for the sortase activity (Ton-That et al. 1999); and recognize their substrate proteins via a common C-terminal pentapeptide sequence, which acts as a cell wall sorting signal. Substrate proteins are not directly transferred to the cell wall, but to the peptidoglycan intermediate lipid II. So far, more than 700 putative sortase substrates encoded by more than 50 different prokaryotic genomes have been identified. The majority of these proteins are anchored by a sortase named SrtA originally identified in *Staphylococcus aureus *(Mazmanian et al. 1999). The number and types of proteins anchored by SrtA are predicted to vary from two in *B. subtilis *to up to 43 in *Listeria monocytogenes *(Boekhorst et al. 2005). These proteins are recognized in most cases by the pentapeptide sorting signal LPXTG (Fischetti et al. 1990).

Two putative sortase homologues of *B. subtilis *are YhcS and YwpE (Comfort and Clubb 2004; Pallen et al. 2001). YhcS encodes a protein of 198 amino acids carrying a transmembrane anchor at its N-terminus and the active site motif (LxTC). YwpE encodes a small protein of 102 amino acids with the LxTC motif at the C-terminus, but it has no signal peptide at the N-terminus (Clancy et al. 2010; Tjalsma et al. 2000). YhcS has been classified in group SrtD sortases, but there is no clear experimental evidence that class SrtD sortases recognize and anchor proteins on the surface of Gram-positive bacteria (Dramsi et al. 2005).

*B. subtilis *also encodes two potential sortase substrates, YfkN and YhcR, encoded by the *yfkN *and *yhcR *genes (Boekhorst et al. 2005; Comfort and Clubb 2004). Instead of the LPXTG motif, YfkN contains the potential sorting signal LPDTA and YhcR the sequence LPDTS. YfkN exhibits 2', 3' cyclic nucleotide phosphodiesterase and 2' (or 3') nucleotidase and 5' nucleotidase activities, a trifunctional nucleotide phosphoesterase (Chambert et al. 2003). YhcR appears to have 5'-nucleotidase activity, a property shared by LPXTG proteins from several other bacteria (Pallen et al. 2001). Its N-terminal end (residues 1 to 46) contains a signal peptide that is predicted to direct secretion by the twin-arginine translocation pathway, while the C-terminal end is a typical Gram-positive anchor (Oussenko et al. 2004). Furthermore, *yhcR *is located adjacent to *yhcS *on the *B. subtilis *chromosome, one of the two sortase-like proteins in *B. subtilis*. In addition, recent analysis has shown that YfkN and YhcR could accumulate in the culture medium when investigated in *B. subtilis *cells carrying null alleles in *yhcS *and *ywpE*. Therefore, YfkN and YhcR could, in principle, be sorted to the cell wall by the *B. subtilis *sortase homologues YwpE and/or YhcS (Westers 2004).

Despite being intensively studied as a model organism and possessing two sortase-like proteins, there is no direct published evidence that *B. subtilis *might decorate its surface with sortase-dependent proteins covalently linked to the peptidoglycan. In an effort to develop *B. subtilis *as a cellular chip, we have already established a system to immobilize proteins on the surface of a *B. subtilis *strain expressing *L*. *monocytogenes srtA *(Nguyen and Schumann 2006). This work aims to analyze expression of the two putative sortases, YwpE and YhcS, and the two surface proteins, YhcR and YfkN, in order to extend tools to display proteins on the surface of any *B. subtilis *wild type strain using its own sortase(s).

## Materials and methods

### Bacterial strains and culture conditions

The bacterial strains and plasmids used are listed in Table 1. *E. coli *strain DH10B (Stratagene) was used as recipient in all cloning experiments. The *B. subtilis *strain 1012 was used for the construction of new strains and as a template for PCR if not mentioned otherwise. Cells were routinely grown aerobically in Luria-Bertani (LB) broth at 37°C, and antibiotics were added as appropriate (ampicillin at 100 μg/ml, chloramphenicol at 10 μg/ml, erythromycin at 1 or 100 μg/ml, and neomycin at 10 μg/ml).

**Table 1:** Bacterial strains and plasmids used

***B. subtilis***	**Description**	**Reference**
1012	*leuA8 metB5 trpC2 hsdRM1*	(Saito et al. 1979)
NDH03	WW02 with *srtA *gene of *Listeria monocytogenes *integrated at the *lacA *locus	(Nguyen and Schumann 2006)
NDH20	1012 carrying pNDH26 inserted into the chromosome	This work
NDH21	1012 carrying pNDH27 inserted into the chromosome	This work
NDH30	1012 *yhcS *:: *neo *(Neo^R^)	This work
NDH31	1012 *yhcS *:: *neo*, *ywpE *:: *erm *(Neo^R^, Erm^R^)	This work
NDH32	WB800 *yhcs:: neo*, *ywpE::erm *(Neo^R^, Erm^R^, Cm^R^)	This work
SZ59	1012 *yhcS *:: *cat *(Cm^R^)	This work
SZ60	1012 *ywpE *:: *erm *(Em^R^)	This work
WB800	*nprE aprE epr bpr mpr *:: *ble nprB *:: *bsr *Δ*vpr wprA *:: *hyg *(Cm^R^)	(Wu et al. 2002)
WB800N	WB800 pB-*cat*5-*neo*-*cat*3 (Neo^R^)	This work
		
Plasmids		
pBluescript II KS		Stratagene
pMUTIN4	Integration vector carrying *lacZ *and *erm*	(Vagner et al. 1998)
pNDH19	P*xylA*-*amyQ*-*fnbpB123*	(Nguyen and Schumann 2006)
pNDH26	pMUTIN4 carrying 5' end of *yhcS*	This work
pNDH27	pMUTIN4 carrying 5' end of *ywpE*	This work
pNDH33	Expression vector carrying P*grac *and Cm	(Phan et al. 2006)
pNDH33-*yhcS*	pNDH33 carrying *yhcS *(P*grac*-*yhcS*)	This work
pNDH33-*ywpE*	pNDH33 carrying *ywpE *(P*grac*-*ywpE*)	This work
pNDH33-*ywpE-yhcS*	pNDH33 carrying *ywpE-yhcS* (P*grac*-*ywpE-yhcS*)	This work
pNDH37	P*gra*c with signal sequence of *amyQ*	(Phan et al. 2006)
pNDH37-*amyQ*	P*grac *with full length of *amyQ*	(Phan et al. 2006)
pNDH88	pHT01 with *amyQ*	This work
pNDH89	*yhcR123 *translationally fused to *amyQ *	This work
pNDH90	*yfkN123 *translationally fused to *amyQ*	This work
pHT01	Expression vector carrying P*grac *and Cm	(Nguyen et al. 2007)

### Construction of strains NDH20 and NDH21

**Figure 1:**
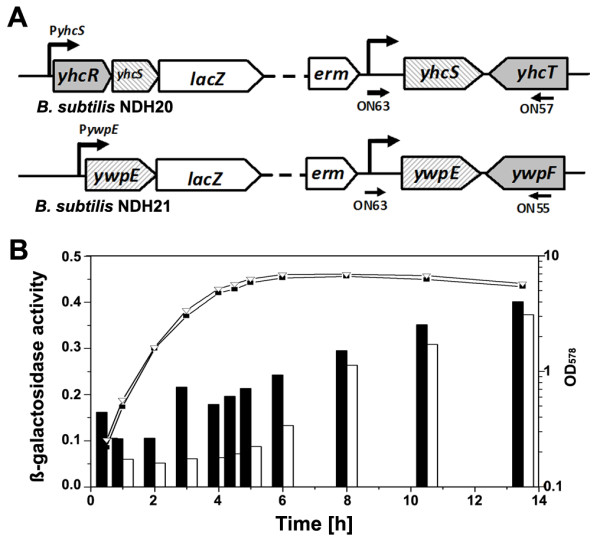
**Transcriptional fusion of the *lacZ *reporter gene to the *yhcS *and *ywpE *promoters**. (A) Schematic representation of transcriptional fusions between the promoters of *yhcS *and *ywpE *and the *lacZ *reporter gene. (B) Cells containing the fusions were grown in LB medium at 37°C, and aliquots were taken at the time points indicated for determination of the OD_578 _and for measuring the β-galactosidase activity. Strains NDH20 ('black square' and closed bars) and NDH21 ('white triangle' and open bars).

To measure expression of the *yhcS *and *ywpE *genes, transcriptional fusions between their promoter regions and the *lacZ *reporter gene were constructed. The 5' coding region of *yhcS *including the start codon was amplified using the primers ON59 and ON60 (Table 2), treated with *Eco*RI and *Bam*HI and ligated into the integration vector pMUTIN4 (Vagner et al. 1998), cleaved with the same enzymes resulting in pNDH26. In a second experiment, the complete *ywpE *gene including its start codon was amplified using the ON61/ON62 primer pair and inserted into pMUTIN4 yielding pNDH27. Both plasmids were transformed into *B. subtilis *1012 resulting in the strains NDH20 and NDH21, respectively (Figure 1). Correct integration at the *yhcS *locus was confirmed by PCR using ON57 and ON63 and at the *ywpE *locus using ON55 and ON63 (Figure 1A). These PCR products were verified by sequencing using ON63. One correct transformant each was kept for further studies.

**Table 2:** Oligonucleotides used

**Name**	**Sequence (5' to 3')**	**Description**
ON29	GGCCATGGATCCATGATTCAAAAACGAAAGCGGACAG	5' end of *amyQ*
ON42	GGCCATGACGTCTTTCTGAACATAAATGGAGACGGAC	3' end of *amyQ*
ON47	GGCCATGACGTCTTGGAAGCGACAGTTGAGTACG	5' end of *yhcR*
ON48	GAATAAGATATCTCACGTTCTGGAGGCGCTCCT	3' end of *yhcR*
ON49	GGCCATGACGTCCGCATGTTTGATATTGAAGAAGC	5' end of *yfkN*
ON50	AGCAGCGATATCTTATGCCTGATTCGCTCTATTCTG	3' end of *yfkN*
ON54	GGCCATTTCGAAGACCTCTTTAGCTCCTTGGAAGC	3' end of *erm*
ON55	GACCTGAATGTGGAACGAGTGGAC	5' end of *ywpF*
ON56	GGCCATTTCGAACCGACTGTAAAAAGTACAGTCGGCA	3' end of *cat*
ON57	CGTCTTGATCAGGATACATCTGGC	5' end of *yhcT*
ON58	GAGAGCCATAAACACCAATAGCCTT	5' end of *neo*
ON59	GGCCATGAATTCAAAGGAGGAACTCCAGAACGTGAAAAAAGTTATTC	5' end of *yhcS*
ON60	CTAATACGACTCACTATAGGGAGAGGATCCCGACACCTTTTTCTAAATCA	3' end of *yhcS*
ON61	GGCCATGAATTCAAAGGAGGAACAACAATGCGCCGGGATCA	5' end of *ywpE*
ON62	CTAATACGACTCACTATAGGGAGAGGATCCTCTTCGTGCTTCACTCTTGC	3' end of *ywpE*
ON63	TCTACATCCAGAACAACCTCTGC	5' end of P*spac*
ON64	GGCCATAGATCTATGCGCCGGGATCAAAAAATG	5' end of *ywpE*
ON65	GGCCATAGATCTATGAAAAAAGTTATTCCACTATTCATCATTGC	5' end of *yhcS*
ON66	GGCCATAGATCTAGAATGAAGAAAAGCCGCAGGCACT	3' end of *yhcS*
ON67	CCAGAGATCTCAAAGGAGGAACTCCAGAACGTGAAAAAAGTTATTC	5' end of *yhcS*
ON68	AGTAAAGTTATCGGAATCGACTTAG	5' end of *dnaK*
ON69	CTAATACGACTCACTATAGGGAGAAAAGTATGCAGGAACTGTGAT	3' end of *dnaK*

Restriction sites used for cloning are underlined

### Construction of strains SZ59, SZ60, NDH30, NDH31 and NDH32

**Figure 2:**
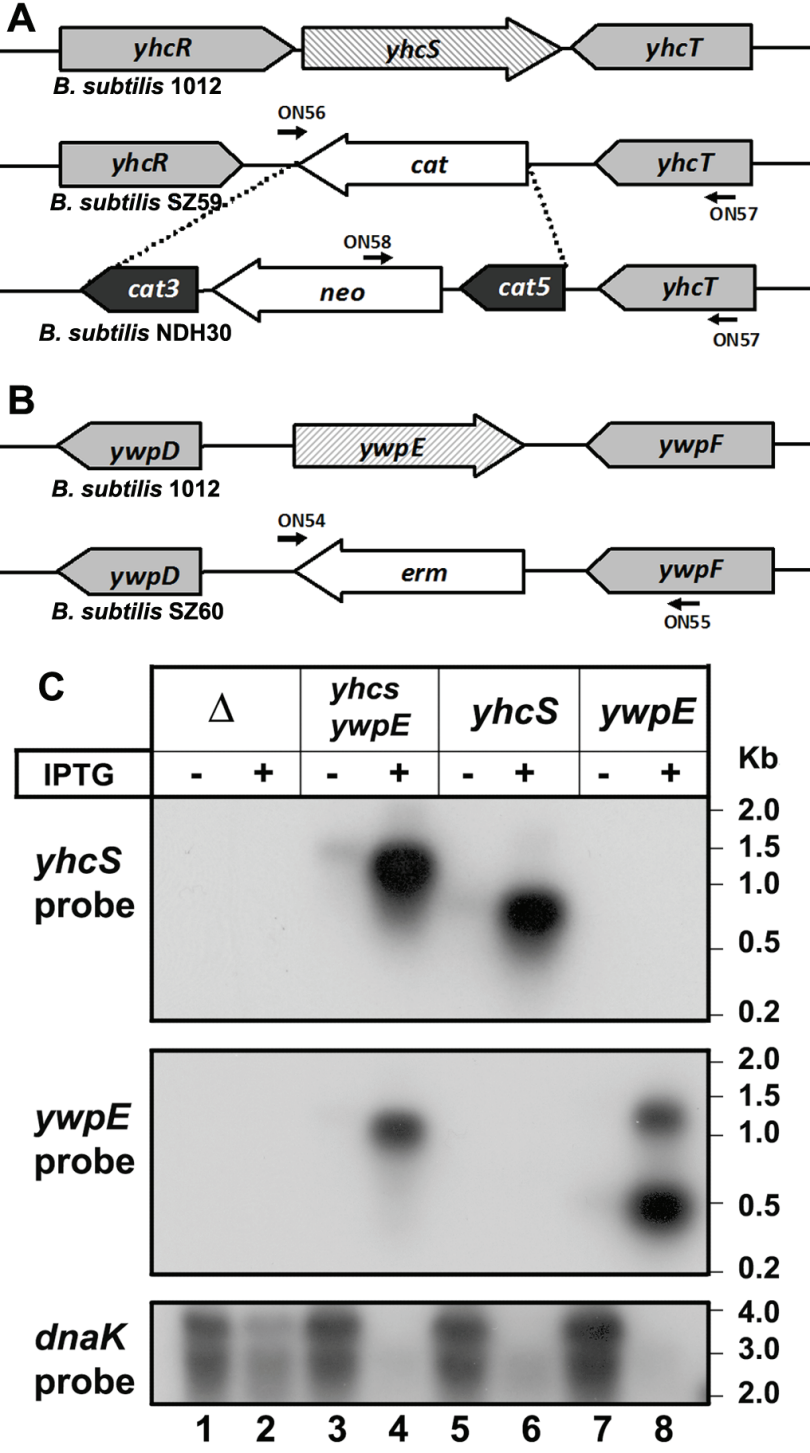
**Expression of *yhcS *and/or *ywpE *in strain NDH31 (Δ*yhcS *and Δ*ywpE*) from plasmids by Northern blot**. (A, B) Schematic representation of chromosomal regions of the knockout strains SZ59, SZ60 and NDH30. The positions of ONs used for verification of the null alleles by PCR are indicated. Three pairs of primers have been used: ON54 and ON55 specifically recognize chromosomal DNA of strain SZ60 (1617-bp PCR product), ON56 and ON57 strain SZ59 (1486-bp PCR product) and ON57 and ON58 strain NDH30 (1602-bp PCR product). (C) Expression of *yhcS *and/or *ywpE *in strain NDH31 with different plasmids (pNDH33, pNDH33-*yhcS*, pNDH33-*ywpE *and pNDH33-*ywpE*-*yhcS*. Either a total of 0.25 μg (lanes 4, 6 and 8) or 5 μg of RNA (lanes 1, 2, 3, 5, 7) were loaded per lane. RNA markers are indicated on the right margin.

To inactivate the genes coding for the two putative sortases, their coding sequences were replaced by two different antibiotic resistance markers. To obtain this goal, *yhcS *was replaced by a chloramphenicol resistance marker resulting in strain SZ59 (*yhcS*::*cat*) and *ywpE *by an erythromycin resistance marker (SZ60: *ywpE*::*erm*) as shown in Figure 2A and 2B. To be able to use these knockout strains with plasmids that carry a chloramphenicol resistance gene, the *cat *cassette in strain SZ59 was replaced by a *neo *cassette. First, the *cat*5-*neo*-*cat*3 cassette was cloned into plasmid pBluescript II KS resulting in plasmid pB-*cat*5-*neo*-*cat*3. This plasmid was treated with *Pvu*II and then transformed into strain SZ59, neomycin-resistant colonies were screened for chloramphenicol sensitivity, and correct integration at the *cat *cassette was confirmed by PCR using ON57 and ON58 (data not shown), and one transformant was kept for further studies (NDH30). Second, chromosomal DNA of strain SZ60 was transformed into the strain NDH30; recombinants were selected on LB plates containing erythromycin and neomycin. Correct integration at the *ywpE *locus was confirmed by PCR using ON54 and ON55, resulting in strain NDH31. Strain NDH32 was generated by transformation of chromosomal DNA of NDH31 into WB800, followed by selection for chloramphenicol, neomycin and erythromycin resistance (strain NDH32).

### Construction of *B. subtilis *strain WB800N

WB800 (Wu et al. 2002) is an eight-fold protease-deficient *B. subtilis *strain that is used for the production of secreted heterologous proteins. This strain is resistant to chloramphenicol. To be able to use it with plasmid pNDH33 derivatives all carrying a chloramphenicol resistance gene (Phan et al. 2006), a *neo *cassette was inserted in the middle of *cat *cassette resulting in strain WB800N. The *Pvu*II-treated plasmid pB-*cat*5-*neo*-*cat*3 was transformed into WB800 and plated on indicator medium, calcium caseinate plates with neomycin. Colonies without halos (as compared with strain 1012) were checked for sensitivity to chloramphenicol and resistance to neomycin. One transformant was kept for further study (WB800N).

### Construction of plasmids able to overexpress the two putative sortases separately and together

To be able to overexpress *yhcS *and/or *ywpE *in *B. subtilis *under the control of the IPTG-inducible promoter P*_grac _*(Phan et al. 2006), three different plasmids were constructed. First, the coding sequence of the *ywpE *gene including its start codon was amplified by PCR using ON64 and ON62, the amplicon was cleaved with *Bam*HI and *Bgl*II and ligated into pNDH33 (Phan et al. 2006) at its unique *Bam*HI site resulting in pNDH33-*ywpE*. The *ywpE *gene was transcriptionally fused to P*_grac _*and a strong ribosome-binding site (RBS) present on pNDH33. Next, gene *yhcS *was amplified using ON65 and ON67 containing its own RBS; the PCR product was then cleaved with *Bgl*II and ligated into pNDH33-*ywpE *resulting in pNDH33-*ywpE*-*yhcS*. The gene *yhcS *was also amplified using ON65 and ON66, the amplicon was treated with *Bgl*II and ligated into pNDH33 at its unique *Bam*HI site resulting in pNDH33-*yhcS*.

### Construction of plasmids pNDH88, pNDH89 and pNDH90

In order to study whether the putative *B. subtilis *sortases could recognize potential sorting sequences, two plasmids that allow anchoring of *amyQ *coding for an α-amylase (Palva 1982) on the cell wall were constructed. In a previous report, it has been suggested that a 123-amino-acids spacer between AmyQ and the sorting sequence is optimal to anchor AmyQ on the cell wall (Nguyen and Schumann 2006). Therefore, plasmids were generated, in which *amyQ *was translationally fused to the putative sorting sequences with the 123-amino-acids spacers encoded by *yhcR *(YhcR123) and *yfkN *(YfkN123) under the control of the IPTG-inducible promoter P*grac*. First, the *amyQ *gene was generated by PCR using pKTH10 (Palva 1982) as template together with ON29 and ON42, the amplicon was treated with *Bam*HI and *Aat*II and ligated into pHT01 (Nguyen et al. 2007) cut with the same enzymes resulting in pNDH88. Next, the coding regions of the 3' ends of *yhcR *and *yfkN *including the sorting motif and the additional 123 codons, the spacer regions, were amplified using ON47/ON48 and ON49/ON50, respectively. The amplicons were cleaved with *Aat*II and *Eco*RV and inserted into pNDH88 treated with *Aat*II and *Sma*I resulting in pNHD89 and pNDH90, respectively.

### Determination of sortase-dependent cell wall proteins

**Figure 3:**
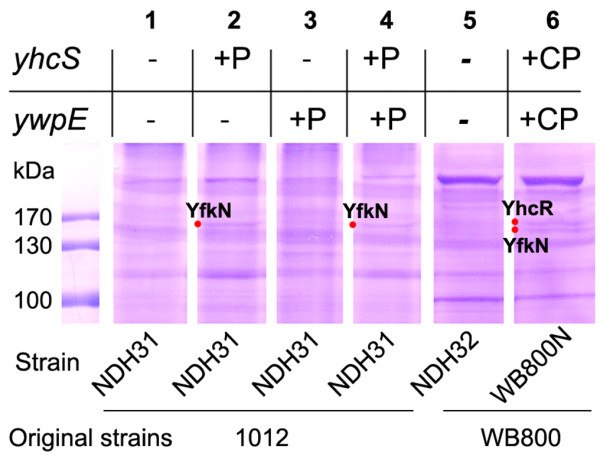
**Protein patterns of the putative sortase knockout strains**. Samples were collected 8 h after induction. The cells were sonicated, followed by intensive washing and lysozyme treatment. Samples for SDS-PAGE and Coomassie blue staining were prepared as described in Materials and methods. The following strains have been analyzed: 1, NDH31/pNDH33 (Δ*yhcS *Δ*ywpE*); 2, NDH31/pNDH33-*yhcS *(Δ*ywpE*) (+P); 3, NDH31/pNDH33-*ywpE*; 4, NDH31/pNDH33-*ywpE*-*yhcS*; 5, NDH32 (Δ*yhcS *Δ*ywpE*) derived from WB800 and 6, WB800N/pNDH33-*ywpE*-*yhcS *carrying *yhcS *and *ywpE *both on the chromosome and on the plasmid (+CP) were investigated. Strain NDH31 was derived from *B. subtilis *1012, and NDH32 and WB800N were derived from *B. subtilis *WB800. The size of molecular weight standards is indicated on the left margin.

The *B. subtilis *strains were inoculated to an OD_578 _of 0.05 - 0.08 in LB medium and grown at 37°C in a shaking water bath. After 1 h of growth, 0.1 mM IPTG was added to induce expression of *yhcS *and/or *ywpE *and cells corresponding to 200 OD_578 _units were collected after about 8 h. After sedimentation by centrifugation, the cells were resuspended in 1.5 ml of water (final volume) containing a cocktail of protease inhibitors (Roche Diagnostics), 2 mM EDTA and 100 mg/ml DNase I and disrupted by sonication (12 W, 10 × 30 pulses with 30 sec intervals) on ice. The unbroken cells were removed by low-speed centrifugation (980 × g) at 4°C for 10 min. The supernatants were then centrifuged at higher speed (21 000 × g) at 4°C for 15 min to obtain a pellet containing the envelope material. These pellets were washed three times in water containing protease inhibitors. Finally, the pellets containing peptidoglycan with cell wall proteins were resuspended in 100 μl of lysozyme (1 mg/ml), incubated at 37°C for 45 min and shaken occasionally. Samples were mixed with 3 × loading buffer and applied to SDS-PAGE (Figure 3). The target protein bands were extracted from the gel, and proteins were identified by MALDI-TOF mass spectrometry.

### Enzyme assays

*B. subtilis *strains NDH20 and NDH21 (Figure 1A) containing the transcriptional fusions P*yhcS*-*lacZ *and P*ywpE*-*lacZ *were grown in LB medium at 37°C. When an OD578 of 0.6 was reached (set as t = 0) and samples were collected at the indicated time points. β-Galactosidase activity assays were performed in triplicate with soluble extracts using α-nitrophenyl-β-D-galactoside as substrate (Miller 1972) and yielded comparable results. The activities of one representative experiment are presented. β-Galactosidase activities are given in units, where one unit is defined as ΔA_405 _min^-1 ^× OD_578_^-1 ^× 10^-3^, in which OD_578 _is the optical density of the growth culture.

To measure α-amylase activity, the *B. subtilis *strains 1012/pNDH37, 1012/pNDH37-*amyQ*, NDH30/pNDH89, NDH03/pNDH19 and 1012/pNDH89, three different clones for each strain were grown in LB medium containing chloramphenicol (10 μg/ml) at 37°C. When the OD_578 _of the cultures reached to mid-log phase (OD_578 _0.6), 0.5 mM IPTG and 0.5% xylose were added to all cultures to induce production of sortase A in the NDH03 strains, amylase (AmyQ, from pNDH37-*amyQ*) and the hybrids AmyQ-FnbpB123 (from pNDH19) and AmyQ-YhcR123 (pNDH89). Cells were separated from the growth medium by centrifugation, washed twice with the medium and once with PBS buffer (pH 7.4) and finally resuspended in PBS buffer. Cells corresponding to OD_578 _of 0.2 in 100 μl were used to measure the α-amylase activities. As a control, the enzymatic activity secreted in the supernatant from the strain 1012/pNDH37-*amyQ *was also determined and was set at 100%.

### RNA extraction and Northern blot analysis

*B. subtilis *cells were grown and induced as described under enzyme assays. Strains containing plasmids pNDH33-*yhcS *and pNDH33-*ywpE*-*yhcS *were induced by 0.1 mM IPTG, and the cells were killed by addition of "killing buffer" (5 mM MgCl_2_, 20 mM NaN_3_, 20 mM Tris-HCl; pH7.5). Total RNA was extracted using the protocol for isolation of RNA from yeast with modification (Robert 1998). The cell walls were digested by addition of lysozyme (1 mg/ml) on ice and the samples were then heated at 95°C for 5 min before addition of phenol. The RNA concentration was measured at 280 nm and 10 μg of total RNA was loaded in each well. Northern-blot analyses were performed as described (Roche Company 2003) with antisense RNAs produced against the putative sortase mRNAs. Hybridizations specific for the putative sortase genes were carried out with digoxigenin (DIG)-labelled riboprobe RNAs synthesized *in vitro *with T7 RNA polymerase from PCR products equipped with a promoter recognized by that polymerase (DIG RNA labelling kit; Roche Diagnostics, Mannheim, Germany). Pairs of primers ON59/ON60 and ON61/ON62 were used to amplify an internal part of the *yhcS *and the complete *ywpE *gene, respectively. The ON68/ON69 primers were used to amplify *dnaK *used as a loading control (Homuth et al. 1999).

## Results

### Natural expression of *yhcS *and *ywpE*

*B. subtilis yhcS *codes for a putative sortase of 198 amino acids with one predicted transmembrane domain, while *ywpE *encodes a predicted cytoplasmic protein of only 102 amino acid residues (Comfort and Clubb 2004; Pallen et al. 2001). The latter exhibits 23% sequence identity with the C-terminal domain of SrtA. To follow expression of the two genes during growth, each promoter was fused to the *lacZ *reporter gene (Figure 1A), and the β-galactosidase activity was measured during growth. First, expression of the *ywpE *gene turned out to be lower than that of the *yhcS *gene during exponential and early stationary phase, but both activities were comparable during late stationary phase (Figure 1B). Second, expression of both genes increased over time to be highest during late stationary phase. We conclude from this result that both putative sortase genes are preferentially expressed after cells have entered the stationary phase.

**Figure 4:**
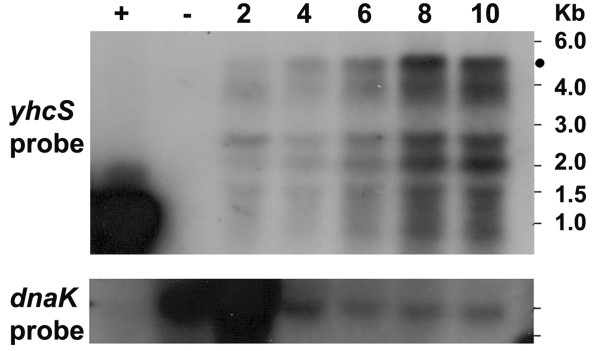
**Detection of the expression of *yhcS *by Northern blot**. Three different *B. subtilis *strains were grown in LB medium and aliquots were analysed by Northern blot using either *yhcS *(upper panel), *ywpE *(middle panel) or *dnaK *antisense RNA (lower panel, loading control). +, strain NDH31/pNDH33-*ywpE*-*yhcS *where both genes coding for putative sortases were artificially expressed from an IPTG-dependent promoter; -, strain NDH31 where both putative sortase genes have been deleted. Lanes 2 to 10, aliquots from wild type strain 1012 were withdrawn at 2, 4, 6, 8 and 10 h after inoculation.

Next, we analyzed transcription of the two genes in the *B. subtilis *1012 wild type strain directly by Northern blot to confirm these results. Total RNA was isolated at different time points during growth and hybridized against gene-specific DIG-labelled anti-sense RNA. A transcript of about 4.5 kb could be detected after 6 h of growth with a further increase at 8 h (Figure 4). The 4.5-kb transcript corresponds by size to the bicistronic *yhcR*-*yhcS *operon (Price et al. 2005). The smaller bands below the 4.5-kb transcript most probably represent processing or/and degradation products. The failure to detect a *ywpE*-specific transcript even when using a large amount of RNA (30 μg) could indicate instability (data not shown). When both genes were expressed artificially from an IPTG-inducible promoter, their bicistronic transcript was produced in high amounts (Figure 4, left lane) indicating full stability under these conditions. In conclusion, putative sortase *yhcS *gene is expressed preferentially at the late stationary phase while *ypwE *expression could not be detected in the Northern blot analysis. This might point to a role of at least one of these two enzymes (YhcS) in anchoring proteins during stationary phase to the cell wall.

### Search for *yhcS *and/or *ywpE*-dependent surface proteins

To identify putative sortase-dependent substrate proteins, the three knockout strains SZ59 (Δ*yhcS*), SZ60 (Δ*ywpE*) and NDH31 (Δ*yhcS *and Δ*ywpE*) were constructed (Figure 2A and 2B). All three mutant strains together with the isogenic wild-type strain were incubated in LB medium for 8 h corresponding to the late stationary phase and analyzed for the presence of cell wall anchored proteins as described under Materials and methods. No difference in the protein pattern could be detected (data not shown). We conclude that the amount of proteins anchored is not sufficient to be detected either due to the low amount of sortase enzymes or due to these two enzymes, or due to a mixture of both. Therefore, we decided to repeat this experiment with strains, where either *ywpE *or *yhcS *or both genes could be expressed together using the IPTG-inducible promoter, P*grac *from plasmid pNDH33. Expression of these genes was analyzed by Northern blot. While induced expression of *yhcS *and *ywpE *yielded the expected RNAs of about 1 and 0.5 kb, respectively, the artificial bicistronic operon led to the detection of an RNA larger than 1 kb (Figure 2C). These experiments clearly demonstrate that both genes can be expressed if fused to a strong promoter.

Interestingly, strains that restored expression of *ywpE *and/or *yhcS *exhibited two YhcS-dependent proteins with molecular weights between 130 kDa and 170 kDa which appeared in the strains that express *yhcS *(Figure 3, lanes 2 and 4). In addition, when the strain WB800N, deficient for eight different proteases (Wu et al. 2002), carrying plasmid pNDH33-*ywpE*-*yhcS *was analyzed several bands became visible. Among them a band running with a molecular mass of 140 kDa seems to be a doublet (Figure 3, lane 6). These protein bands were then extracted and proteins were determined by MALDI-TOF mass spectrometry. As we expected one of these proteins is YhcR and the other is YfkN, both containing the secretional sequence and potential sorting signal.

### Displaying AmyQ on the surface using the sorting sequences

**Figure 5:**
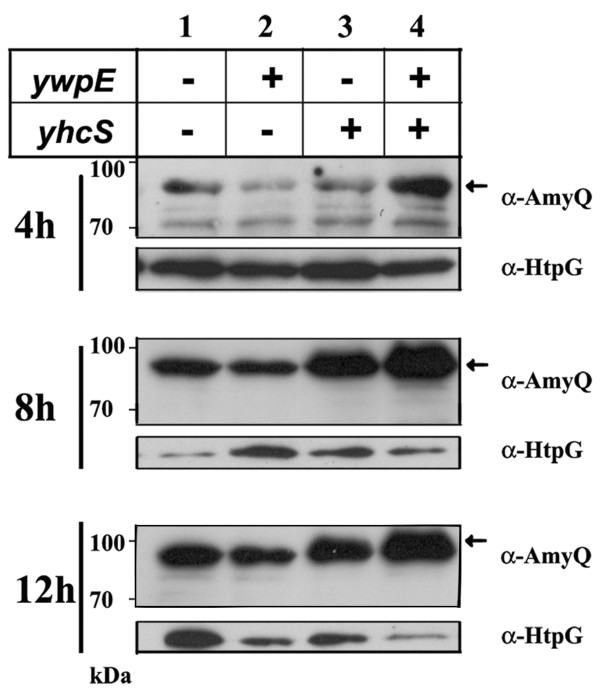
**Detection of α-amylase anchored on the cell wall of four different strains using either the YhcR123 sorting sequence with the 123-aa spacer by Western blot**. All *B. subtilis *strains were inoculated to an OD_578 _of 0.05 - 0.08 in LB medium. After 1 h of growth, 0.1 mM IPTG was added to induce expression of the hybrid *amyQ *gene and cells were collected 4, 8 and 12 h after further inoculation. Equal amounts of cells were treated with lysozyme to release the anchored α-amylase. The samples were applied to SDS-PAGE and Western blot as described (Nguyen and Schumann 2006). Strains NDH31 (Δ*ywpE*, Δ*yhcS*; lane 1), NDH30 (*ywpE*^+^, Δ*yhcS*, lane 2) SZ60 (Δ*ywpE*, *yhcS*^+^; lane 3) and 1012 (*ywpE*^+^, *yhcS*^+^; lane 4), all of them carrying the plasmid pNDH89 (AmyQ-YhcR123). HtpG, a cytoplasmic protein, was used as loading control for the proteins released from the cytoplasm.

**Figure 6:**
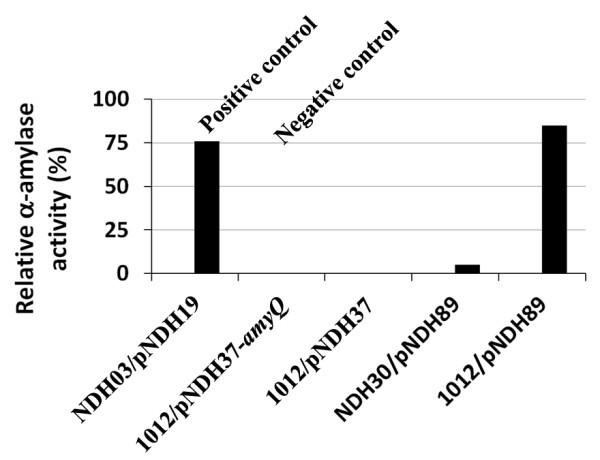
**α-Amylase activities in the presence and absence of potential sortases**. The following strains were analyzed: 1012/pNDH37 (basic expression vector with the IPTG-inducible promoter P*grac *and the signal sequence of *amyQ*), 1012/pNDH37-amyQ (secretes α-amylase into the medium), NDH03/pNDH19 (contains the xylose-inducible *srtA *of *L. monocytogenes *and *amyQ *fused to the sorting sequence of FnBPB), NDH30 (Δ*yhcS*)/pNDH89 (AmyQ-YhcR123) and 1012/pNDH89. Cells were grown to the mid log-phase and then, 0.5 mM IPTG and 0.5% xylose were added into all five cultures to induce production of sortase A (strain NDH03), wild-type amylase (pNDH37-*amyQ*) and hybrid α-amylase (from pNDH19 and pNDH89). Samples were collected after 4 h of induction and the cells were separated from the growth medium by centrifugation. α-Amylase activities were determined with whole cells that the number of cells are identical in all probes and with the supernatant from strain 1012/pNDH37-*amyQ*. The activities were presented as relative activity (%), where the activity measured with the supernatant from 1012/pNDH37-*amyQ *was set at 100%.

Next, we asked whether the two putative sortases YhcS and YwpE could anchor the proteins YhcR and YfkN on the surface of *B. subtilis *using their sorting signal. We fused the *amyQ*-encoded α-amylase to the potential sorting signals of the two proteins together with an 123-amino-acid spacer region resulting in pNDH89 (AmyQ-YhcR123) and pNDH90 (AmyQ-YfkN123). These plasmids were transformed into strains SZ60 (Δ*ywpE*), NDH30 (Δ*yhcS*), NDH31 (Δ*yhc*, Δ*ywpE*) and 1012. To determine the amylase activity on the cell surface, strain NDH03/pNDH19 that has been described to immobilize amylase on the surface (Nguyen and Schumann 2006) and strain 1012/pNDH37-a*myQ *(Phan et al. 2006) that secretes the amylase into the culture medium were used as positive and negative control, respectively. Cells of these strains were grown as described under Materials and methods for Western-blot (Figure 5), and samples were collected at the appropriate time points for measuring the amylase activities (Figure 6).

When the α-amylase carrying the YfkN123 sorting sequence was tested, anchored protein was hardly detected in the strain expressing either *yhcS *or *yhcS *and *ywpE *8 h after induction (data not shown). When the YhcR123 motif was tested, a strong anchoring occurred in the presence of YhcS with some further increase upon additional synthesis of YwpE (Figure 5, 8 h and 12 h). But since a substantial amount of AmyQ-YhcR123 is already present in the absence of both putative sortases, these hybrid protein molecules might be retained in the cytoplasmic membrane due to the presence of a hydrophobic region being part of the sorting sequence. The presence of α-amylase attached to cells in the absence of potential sortases could also be observed with whole cells 4 h after induction (Figure 6 NDH30/pNDH89, 5% activity) when compared with the negative controls (0% activity for both 1012/pNDH37 and 1012/pNDH37-*amyQ*), and the positive control (76% activity for NDH03/pNDH19). Additionally, α-amylase activity of the sample that produces both potential sortases and the hybrid protein, AmyQ-YhcR123 (Figure 6, 1012/pNDH89) was as high as the positive control (Figure 6, NDH03/pNDH19); and the same results could be measured for samples collected after 2 h and 8 h of induction (data not shown). This activity-based measurement confirmed that the fusion YhcR123-AmyQ could be displayed on the surface of *B. subtilis*. In summary, these results strongly suggest that the *yhcS *gene codes for a true sortase able to anchor at least YhcR on the cell wall of *B. subtilis *cell and we suggest renaming it to *srtD*. This work could also propose an alternative way to immobilize a heterologous protein on the cell wall of *B. subtilis *using a fusion form of YhcR sorting sequence.

## Discussion

Using bioinformatics tools, two sortase-like genes and two substrate proteins have been identified (Comfort and Clubb 2004; Pallen et al. 2001; Boekhorst et al. 2005). We could show here that the putative sortase genes *ywpE *and *yhcS *are preferentially expressed in the late stationary phase. This finding suggests that these enzymes fulfill their task mainly during that growth phase. Furthermore, we could demonstrate that the two putative sortase-dependent substrate proteins, YfkN and YhcR, can be anchored on the cell wall in the presence of YhcS. In terms of application, this work demonstrated that the YhcR sorting sequence can be specifically used to display heterologous proteins on the cell-wall of *B. subtilis *cells. The *B. subtilis *cell wall contains peptide crosslinks identical to those present in the *L. monocytogenes *cell walls. This suggests that the crosslink of potential surface proteins to the peptidoglycan is formed by the nucleophilic attack of the amino group of m-diaminopimelic acid cross-bridge within the lipid II precursor as in the case of *L. monocytogenes *(Dhar et al. 2000).

Sortases have been used to anchor heterologous proteins on the cell wall of different Gram-positive bacterial species (Wernerus and Stahl 2004; Tsukiji and Nagamune 2009; Clancy et al. 2010). In a previous study, we established a system to display recombinant proteins on the cell wall of *B. subtilis *(Nguyen and Schumann 2006). It consists of the *L. monocytogenes srtA *gene fused to an inducible promoter and inserted into the chromosome and a plasmid-based expression system with the *S*. *aureus *FnBPB sorting signal. Since the AmyQ-FnBPB123 fusion protein could be hardly detected in the absence of the *L. monocytogenes *sortase, it implies that the YhcS sortase could not recognize the sorting signal present in this protein (LPxTG). Here, we show that the YhcS sortase could immobilize YhcR and YfkN with their putative sorting signals LPDTS and LPDTA, respectively. This motif is close to the one recognized by SrtD of *B. anthracis *(LPNTA) (Maresso and Schneewind 2008) and indicates that the YhcS protein really belongs to the group SrtD sortases. Therefore, we suggest renaming the gene *yhcS *into *srtD*.

We are interested in using engineered bacteria as delivery vectors for biopharmaceutical purposes. *B. subtilis *would be an ideal organism since (i) it is a generally recognized as safe (GRAS) organism, (ii) can localize in tumours (Yu et al. 2008) enabling to use engineered *B. subtilis *cells for cancer therapy, and (iii) has a large body of information available to control protein expression in the cytoplasm, on the cell surface and secreted into the culture medium (Pohl and Harwood 2010; Schumann 2007). Different protein expression systems have been developed using small inducer molecules such as xylose (Kim et al. 1996), IPTG (Phan et al. 2010; Nguyen et al. 2005), arabinose (De Lencastre and de Sa-Nogueira 2000), tetracycline (Kamionka et al. 2005), glycine (Phan and Schumann 2007) and lysine (Phan and Schumann 2009). (iiii) Additionally, surface displaying systems are available to immobilize proteins (Nguyen and Schumann 2006) that can bind to the surface of mammalian cells facilitating the internalization of the engineered bacteria (Bierne et al. 2002). Engineered bacteria expressing an appropriate surface protein facilitating their internalization into mammalian cells, furthermore a protein enhancing their survival in the host cells and a functional protein are called cellular chips or microbiorobots. Microbiorobots can be used as a vaccine delivery vector (Paccez et al. 2007) or for the development of a cancer therapy in the near future.

## Competing interests

The authors declare that they have no competing interests.
